# Precision Medicine for Obesity Treatment

**DOI:** 10.1210/jendso/bvaf102

**Published:** 2025-06-05

**Authors:** Maria Antonia Espinosa, Rene de J Rivera Gutierrez, Jose Villamarin, Andres Acosta

**Affiliations:** Precision Medicine for Obesity Program, Mayo Clinic, Rochester, MN 55905, USA; Division of Gastroenterology and Hepatology, Mayo Clinic, Rochester, MN 55905, USA; Precision Medicine for Obesity Program, Mayo Clinic, Rochester, MN 55905, USA; Division of Endocrinology, Diabetes, and Metabolism, Mayo Clinic, Jacksonville, FL 32224, USA; Precision Medicine for Obesity Program, Mayo Clinic, Rochester, MN 55905, USA; Division of Gastroenterology and Hepatology, Mayo Clinic, Rochester, MN 55905, USA; Precision Medicine for Obesity Program, Mayo Clinic, Rochester, MN 55905, USA; Division of Gastroenterology and Hepatology, Mayo Clinic, Rochester, MN 55905, USA

**Keywords:** obesity, precision medicine, multi-omics, obesity phenotypes, obesity treatments

## Abstract

Obesity is a chronic, heterogeneous, and complex disease associated with increased morbidity, mortality, and health-care costs. By 2030, 1 in 2 people in the United States will have obesity. Lifestyle interventions are the cornerstone of obesity management. However, they often fail to achieve clinically significant weight loss, necessitating additional treatments involving pharmacotherapy or procedures. Advancements in obesity pharmacotherapy have improved weight-loss outcomes and reduced associated comorbidities. Despite these advances, variability in response to treatment between individuals is still considerable. This variability reflects limitations in the current “one-size-fits-all” approach to obesity management. A precision medicine approach aims to address this gap by incorporating genetic, physiological, and behavioral characteristics to guide treatment selection and improve outcomes. Advances in multi-omics technologies, such as genomics, proteomics, metabolomics, and microbiome profiling, offer new opportunities to refine patient stratification and identify novel therapeutic targets. These tools may help move obesity care toward a more individualized and mechanism-based approach.

Obesity is a chronic, heterogeneous, and complex disease [1]. The World Obesity Federation projects that the prevalence of obesity will rise from 0.81 billion cases in 2020 to 1.53 billion cases by 2035 [2]. In the United States, 1 in nearly 2 adults are projected to have obesity by 2030 [3]. In 2022, obesity imposed a substantial economic burden on health systems worldwide. Medical expenses accounted for 0.7% to 17.8% of total health expenditures [4]. In various countries, obesity represented 0.5% to 2.4% of the gross domestic product, highlighting the increasing importance of obesity management [4].

The main goal of obesity treatment is to help patients move from habits that promote weight gain to those that support weight loss and prevent weight recurrence, ultimately improving quality of life [5]. However, while essential, lifestyle interventions often fail to produce statistically significant and lasting results. In the Look AHEAD study, which evaluated weight-loss outcomes over an 8-year follow-up period among adults with overweight or obesity and type 2 diabetes, participants in the intensive lifestyle intervention group lost more weight than those receiving standard care during the first year (8.5% vs 0.6%, respectively). However, by year 8, a categorical analysis of the intensive lifestyle intervention group revealed that only 39.3% maintained a greater than 10% weight loss, 25.8% maintained 5% to less than 10%, 20.7% maintained 0% to less than 5%, and 14.2% regained weight above baseline [6]. Moreover, weight-loss interventions, such as antiobesity medications (AOMs), exhibit substantial variability in effectiveness across individuals. The STEP 1 trial, a pivotal study assessing the efficacy of semaglutide 2.4 mg once-weekly for weight loss in adults with overweight or obesity, demonstrated a mean weight loss of 14.9% at 68 weeks [7]. However, in an extension of the STEP 1 trial the SD reported was 9.3%, highlighting the substantial individual variability in weight-loss response [8]. This heterogeneity is also present after bariatric procedures. For example, a prospective study following 418 patients post Roux-en-Y gastric bypass showed that at 12 years, while 93% of patients maintained at least a 10% weight-loss change from baseline, only 40% maintained at least a 30% weight loss [9]. This variability remains a clinical challenge, underscoring the need for enhancing therapeutic strategies in obesity management.

Precision medicine aims to stratify diseases, predict their progression and the development of associated comorbidities, and guide treatment by integrating the latest clinical advancements, measurable behavioral and physiological traits, and insights from -omics assays (eg, genomics, proteomics, transcriptomics, microbiomics) [10]. Precision medicine for obesity aims to understand the mechanisms underlying the complex and heterogeneous nature of obesity. It seeks to optimize the selection of lifestyle interventions, AOMs, and bariatric procedures to reduce variability in weight-loss outcomes.

The review aims to synthesize current research on precision medicine for obesity, highlighting advancements in its application for obesity treatment.

## Precision Medicine

**Figure 1.**
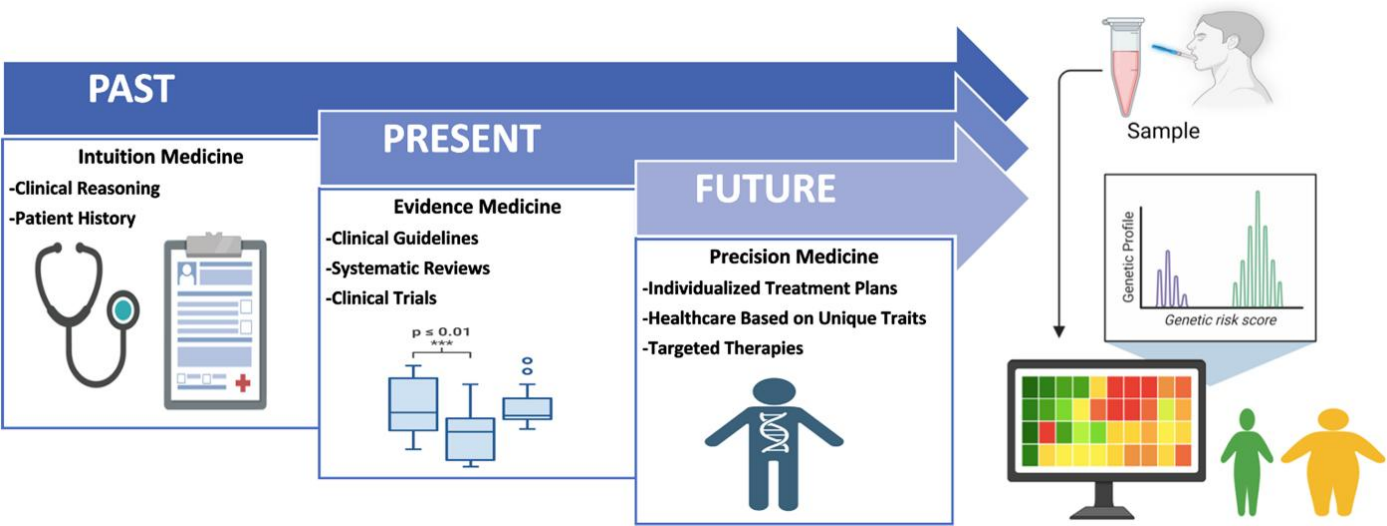
Obesity management: from intuition medicine to precision medicine. Intuition medicine relies on personal judgment, while evidence medicine uses scientific data to guide treatment. Precision medicine tailors care based on individual genetic, environmental, and lifestyle factors.

Medicine has evolved from intuition-based approaches to evidence-based medicine and is advancing toward precision medicine (Fig. 1) [11]. Precision medicine considers individual variability with the potential to develop targeted preventive, diagnostic, and therapeutic strategies to improve disease stratification and treatment effectiveness [12, 13]. Emerging advancements in energy balance regulation, genotyping, multi-omics, and big data analysis may reveal novel signatures or phenotypes predictive of best responders to specific interventions [14]. As this field progresses, it has the potential to improve health outcomes and transform health-care delivery into a more efficient and cost-effective system [15].

## Current Obesity Classifications

Traditional anthropometric classifications of obesity correlate with cardiovascular risk and mortality [16, 17]. However, they fail to elucidate the complexity of obesity pathogenesis. Therefore, an obesity stratification based on mechanistic phenotypes may provide a path to understanding the root cause of obesity at the individual level.

### Body Mass Index

Obesity is traditionally classified using body mass index (BMI), which is the measurement of weight in kilograms divided by height in square meters (kg/m^2^) [18]. Obesity treatment uses standard management guidelines to determine interventions based on BMI categories and associated comorbidities [18, 19]. Table 1 summarizes of the most common guidelines based on BMI.

**Table 1.** Summary of obesity management guidelines by body mass index classification

BMI category	Recommendations	Source
25-29.9, overweight	Overweight without additional risk factors: counsel on preventing further weight gain and advise patients to engage in regular physical activity	AHA/ACC/TOS 2013
	Lifestyle interventions (diet, physical activity, behavior therapy)	AHA/ACC/TOS 2013, AACE/ACE 2016
	Consider adjunctive pharmacotherapy if BMI ≥ 27 with obesity-associated comorbidities*^a^*	AACE/ACE 2016
30-34.9, obesity class 1	Lifestyle interventions (diet, physical activity, behavior therapy)	AHA/ACC/TOS 2013, AACE/ACE 2016
	Consider adjunctive pharmacotherapy	AACE/ACE 2016
35-39.9, obesity class 2	Consider adjunctive pharmacotherapy	AACE/ACE 2016
	Lifestyle interventions (diet, physical activity, behavior therapy)	AHA/ACC/TOS 2013, AACE/ACE 2016
	Offer referral for bariatric surgery consultation and evaluation if comorbidities present*^a^*	AHA/ACC/TOS 2013, AACE/ACE 2016
≥40, obesity class 3	Offer referral for bariatric surgery consultation and evaluation	AHA/ACC/TOS 2013, AACE/ACE 2016
	Consider adjunctive pharmacotherapy	AACE/ACE 2016
	Lifestyle interventions (diet, physical activity, behavior therapy)	AHA/ACC/TOS 2013, AACE/ACE 2016

Table based on AHA/ACC/TOS 2013 [19] and AACE/ACE 2016 [18] recommendations.

Abbreviations: AACE, American Association of Clinical Endocrinologists; ACC, American College of Cardiology; ACE, American College of Endocrinology; AHA, American Heart Association; BMI, body mass index; TOS, The Obesity Society.

^a^Indicators of increased cardiovascular risk (eg, diabetes, prediabetes, hypertension, dyslipidemia, elevated waist circumference, or other obesity-related comorbidities).

While widely used, the BMI classification system has several limitations and important considerations. BMI cannot directly measure body fat mass percentage or account for visceral fat [20]. Therefore, BMI tends to misclassify people with a high muscle-to-fat ratio as individuals with obesity and can overlook metabolic improvements after exercise [21]. In addition, optimal BMI cutoff values can vary significantly between different racial groups, with the Asian population having lower BMI thresholds compared to White individuals [22].

### Waist Circumference and Waist-Hip Ratio

Waist circumference (WC) and waist-hip ratio (WHR) are commonly used to assess central obesity. Table 2 includes a summary of the most used anthropometric indices. Cutoff values for WC have different predictive values for obesity-associated comorbidities depending on the population. For example, an analysis of data from National Health and Nutrition Examination Survey III found that WC cutoffs produced varying predictive values for different cardiovascular disease (CVD) risk factors depending on ethnic groups [25].

**Table 2.** Anthropometric indices of abdominal obesity

Indicator	Cutoff points
BMI [23]	<18.5, underweight
	18.5-24.9, normal range
	25.0-29.9, overweight
	30.0-34.9, obesity class 1
	35.0-39.9, obesity class 2
	≥40, obesity class 3
Waist circumference [24]	>102 cm (M); >88 cm (F)
Waist-Hip ratio [24]	≥0.90 cm (M); ≥0.85 cm (F)

Abbreviations: BMI, body mass index; F, female; M, male.

The WHR is a surrogate marker for central obesity associated with CV comorbidities and all-cause mortality [26-28]. An observational study of 387 672 participants from the UK Biobank found that a higher WHR had a 22% increased risk of all-cause mortality compared to BMI [28].

Although cost-effective, the use of the discussed anthropometric indices has limitations. On one hand, they cannot distinguish between subcutaneous and visceral fat [29]. On the other hand, these indices often underestimate or overestimate disease risk across various ethnic, genetic, age, and sex groups. As such, they have reduced accuracy and applicability in designing tailored obesity management strategies.

### Edmonton Obesity Staging System

The Edmonton Obesity Staging System (EOSS) is a 5-stage obesity risk-stratification system that scores from 0 to 4 (Table 3). It incorporates physical, psychological, and metabolic parameters. Its role in achieving clinically significant weight loss is substantial, as studies have shown its value in guiding treatment decisions and improving outcomes in obesity management. For example, higher EOSS stages increase the risk of complications in bariatric surgery. In a cohort of 9437 participants, complication rates reached 5.3%, with the highest rates in stages 3 (7.8%) and 4 (6.8%) [31]. Another study with 430 238 participants found that EOSS scores independently predicted a 30-day major complication rate of 3.5%. In nonsurgical settings, EOSS also proved to be valuable. An intensive weight-management program achieved clinically significant, greater than 5% weight loss in 50% and 70% of participants in stages 2 and 3, respectively [32 ].

**Table 3.** Edmonton Obesity Staging System

EOSS stage	Description
0	No apparent obesity-related risk factors, physical symptoms, psychopathology, functional limitations, and/or impairment of well-being
1	Presence of obesity-related subclinical risk factors, mild physical symptoms, mild psychopathology, mild functional limitations, and/or mild impairment of well-being
2	Presence of established obesity-related chronic disease, moderate limitations in activities of daily living, and/or moderate impairment of well-being
3	Established end-organ damage such as myocardial infarction, heart failure, diabetic complications, significant psychopathology, significant functional limitations, and/or significant impairment of well-being
4	Severe (potentially end-stage) disabilities from obesity-related chronic diseases, disabling psychopathology, severe functional limitations, and/or severe impairment of well-being

Table modified from Sharma et al (2009) [30].

Abbreviation: EOSS, Edmonton Obesity Staging System

However, while EOSS can be helpful to predict mortality and postoperative complications, its predictive power for long-term weight loss and resolution of comorbidities is limited. Ogassavara et al [33] found no significant correlation between EOSS scores and perioperative outcomes in bariatric surgery patients.

### Obesity Phenotypes by Metabolic Factors

Metabolically healthy obesity (MHO) is an obesity phenotype characterized by an absence or minimal presence of the metabolic dysfunctions typically associated with the components of metabolic syndrome [34]. Individuals with MHO exhibit normal insulin sensitivity, blood pressure within healthy ranges, favorable lipid profiles, and reduced levels of inflammatory markers [35]. On the other hand, metabolically unhealthy obesity (MUO) is an obesity phenotype characterized by significant metabolic dysfunction, including insulin resistance, hypertension, dyslipidemia, and elevated inflammatory markers [36]. Individuals with MUO have twice the risk of developing CVDs, type 2 diabetes, and other obesity-related complications [34].

However, this classification has certain limitations. First, MHO individuals still have a higher risk of comorbidities. A prospective cohort study of 381 363 UK Biobank participants showed that MHO individuals, when compared to metabolically healthy individuals without obesity, had higher rates of developing CVD, type 2 diabetes mellitus, and certain cancers [37]. Second, MHO is not a stable condition and individuals with MHO may transition to MUO over time [38]. Last, individuals with MHO may have other issues associated with obesity such as chronic pain, osteoarthritis, and psychosocial factors that are not captured by this classification [39]. Given these limitations, it is important to consider management for all individuals with obesity to reduce health complications in the future.

### Obesity Phenotypes by Energy Balance and Physiological and Behavioral Traits

**Figure 2.**
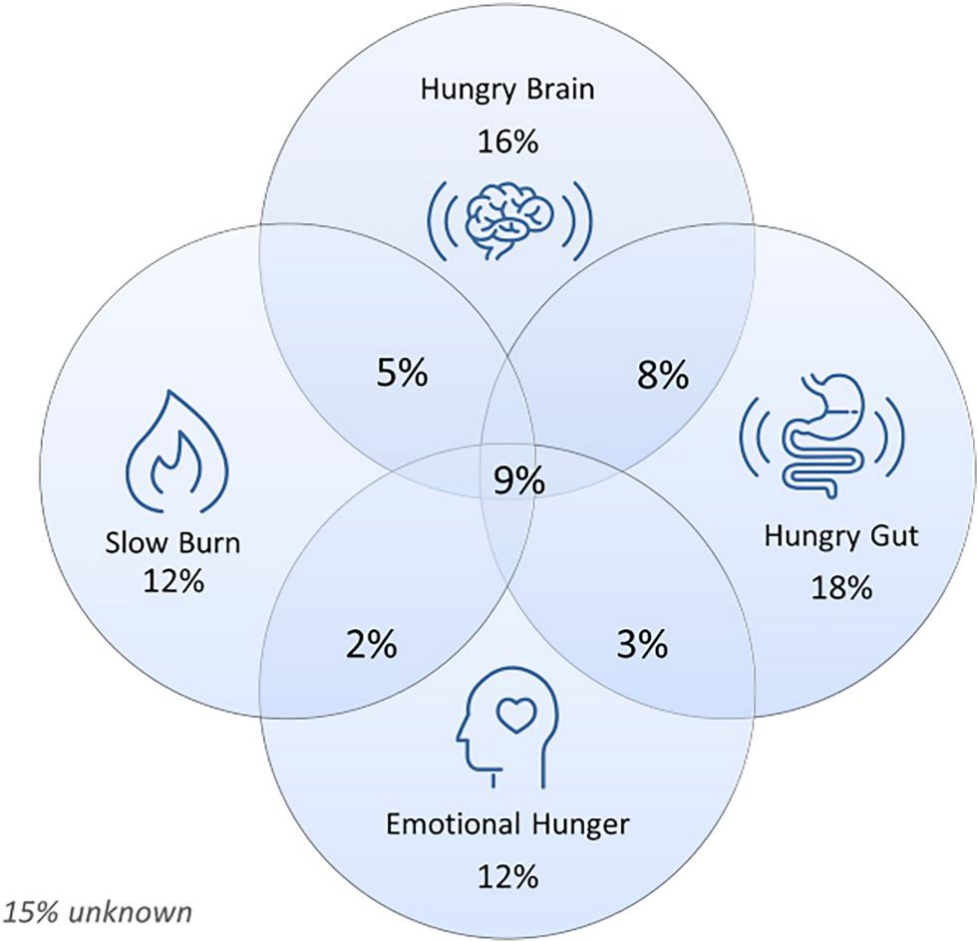
Obesity phenotypes. Four distinct phenotypes: abnormal satiation (hungry brain), abnormal postprandial satiety (hungry gut), low energy expenditure (slow burn), and emotional eating. Retrieved from Acosta et al [40].

We propose analyzing energy regulation in patients with obesity by determining distinct “phenotypes” based on energy balance and physiological and behavioral traits. We describe 4 pathophysiology-informed phenotypes to classify obesity: abnormal satiation, abnormal postprandial satiety, low energy expenditure, and emotional eating (Fig. 2) [40].

These phenotypes are identified after following standard protocol for obesity phenotyping using objective and subjective measurements of energy balance and behavioral traits [40-42]. Patients arrive fasting and undergo indirect calorimetry testing to measure resting energy expenditure. After that, they undergo dual-energy x-ray absorptiometry to analyze body composition. Following a standardized radiolabeled breakfast, we assess for gastric emptying of solids and liquids through scintigraphy. Participants then eat until they feel full during an ad libitum meal to measure the number of calories needed to achieve satiation, using subsequent subjective appetite questionnaires (ie, visual analogue scale scores).

#### Abnormal satiation

Satiation is defined as calories consumed to reach fullness and terminate a meal [43]. It is quantified by the caloric intake required to achieve fullness during an ad libitum meal [42]. Abnormal satiation (referred to as “hungry brain”) is characterized by impaired intrameal inhibition, resulting in the consumption of larger meal sizes [40, 42].

#### Abnormal postprandial satiety

Abnormal postprandial satiety (referred to as “hungry gut”) consists of impaired intermeal inhibition, leading to increased frequency of hunger in between meals [44] and characterized by accelerated gastric emptying [40, 42].

#### Emotional eating

The emotional eating phenotype is marked by a strong hedonic or emotional drive influencing eating behaviors. It is characterized by a tendency to eat in response to positive or negative emotions. Questionnaires like the Three Factor Eating Questionnaire (TFEQ-21) [45] and the Hospital Anxiety and Depression Score (HADS) were used to assess this phenotype in clinical and research settings [40, 42].

#### Low resting energy expenditure

The low resting energy expenditure phenotype (referred to as “slow burn”) consists of lower-than-expected resting energy expenditure, typically assessed through indirect calorimetry compared to expected values calculated by Harris-Benedict equation for basal metabolic rate [40]. In addition to reduced energy expenditure, alterations in physical activity levels and a decrease in diet-induced thermogenesis can individually or collectively contribute to a positive energy balance in individuals with this phenotype, ultimately promoting weight gain [46].

In a cross-sectional study analyzing 464 patients, the presence of multiple phenotypes demonstrated a cumulative effect on weight and BMI. Participants having 2 or more phenotypes exhibited higher body weight (115 kg vs 109 kg) and BMI (40 vs 38) when compared to participants with no more than 1 phenotype [47].

## Precision Medicine for Obesity

There has been substantial progress in obesity interventions; however, the progress seen in medications and surgery is still facing two main limitations. First, there is tremendous heterogeneity in response to different interventions [48]. Thus, it is critical to be able to identify those who are going to respond to each intervention. Second, the heterogeneity of response negatively affects the cost-effectiveness assumptions of obesity interventions. Thus, it is essential to identify responders to maximize the outcomes of each intervention for obesity with the intention to migrate obesity treatment to a more cost-effective, population-based solution. Precision medicine for obesity offers a unique solution for better and more homogenous outcomes.

### Treatment Tailored to Obesity Phenotypes Based on Energy Balance and Behavioral Traits

Treatment tailored to obesity phenotypes has the potential to significantly enhance the current standard of care, offering more personalized and effective management strategies. We propose a working hypothesis to pair obesity interventions with each energy balance and behavioral obesity phenotype. In the next sections we describe treatment strategies for each phenotype and our current working hypothesis of phenotype-guided obesity management (Table 4).

**Table 4.** Working hypothesis of phenotype-guided obesity management

	Hungry brain, abnormal satiation	Hungry gut, abnormal postprandial satiety	Emotional hunger, abnormal emotional eating	Slow burn, abnormal resting expenditure
Lifestyle intervention [41]	• Hungry brain diet	• Hungry gut diet	• Behavioral therapy • Hungry feelings diet	• Intense exercise plan • Slow burn diet
Medication [40, 42, 49, 50]	• Phentermine-Topiramate ER	• Liraglutide • Semaglutide	• Bupropion-Naltrexone SR	• Phentermine
Endoscopy [51-55]	• Vagal nerve block • Endoscopy sleeve gastroplasty	• Intragastric balloons • Intragastric gels		
Surgery [54, 56, 57]	• Laparoscopic sleeve gastrectomy	• Roux-en-Y gastric bypass		

This table categorizes various interventions for obesity based on phenotype types: hungry brain, hungry gut, emotional hunger, and slow burn. Each intervention targets the underlying mechanisms of obesity based on the individual's phenotype, aiming for more personalized, effective treatment strategies.

Abbreviations: ER, extended release; SR, sustained release.

#### Abnormal satiation phenotype

The abnormal satiation phenotype is characterized by requiring more calories at each meal to reach fullness. These patients might benefit from i) decreasing their meal frequency to reduce the daily caloric intake with prolonged fasting periods (time-restricted eating) [58]; ii) increasing dietary nonsoluble fiber (volumetric) [59-61]; and iii) healthy second servings if needed. The purpose is to i) keep the brain hunger center “off” for longer periods of time (as these patients have normal hunger but abnormal satiation) [62]; ii) produce maximal gastric distention and accommodation to induce the sensation of fullness using a volumetric diet [63]; and iii) recognize the need for second servings and suggest healthy second servings, which may also help with reaching satiation [64]. Thus, we recommend a low-calorie volumetric diet, increased fiber, fruits, and vegetables for second servings, with time-restricted eating typically eating during an 8-hour daytime window. We propose that the abnormal satiation phenotype be tailored with time-restricted eating and a volumetric diet to promote satiation [41]. We suggest that phentermine-topiramate extended-release (ER), US Food and Drug Administration (FDA) approved in 2013 for obesity as the medication for this phenotype. This suggestion was based on the observation from a 2-week randomized clinical trial involving 24 patients; those who received phentermine-topiramate ER showed a significant reduction in calorie intake to achieve satiation compared to the placebo group during an ad libitum meal [42], and the ad libitum meal test for satiation was associated with best responders to this medication. In addition, it is also suggested that endoscopic therapies that aim to reduce stomach capacity may be more suitable for patients with an abnormal satiation phenotype [65]. Further research can help to optimize treatment strategies for this specific obesity phenotype.

#### Abnormal postprandial satiety

Abnormal postprandial satiety is characterized by having accelerated gastric emptying and increased postprandial hunger. These patients might benefit from a high-protein diet with protein preloads to increase the early release of gastrointestinal satiety hormones [66, 67]. High-protein diets result in weight loss and improved satiety long term by delaying gastric emptying [67, 68]. Thus, we recommend a low-calorie, high-protein diet with a premeal shake or healthy protein snack, and 3 to 5 meals a day to help with the increase sensation of hunger between meals [41]. We also favor the selection of glucagon-like peptide-1 receptor agonists, which are known to slow gastric emptying as they could potentially restore satiety mechanisms in this subset of patients [49, 50]. In a placebo-controlled randomized trial, liraglutide treatment slowed gastric emptying of solids at 5 weeks (median difference: 70 minutes) and 16 weeks (median difference: 34 minutes) compared to placebo [69].

#### Abnormal energy expenditure

Abnormal energy expenditure is characterized by reduced resting energy expenditure and muscle mass. These patients might benefit from i) a structured exercise plan to increase muscle mass, which accounts for most of the overall energy expenditure ratio [70] and ii) increasing protein intake. There is an excellent dose-response relationship between resistance training and muscle hypertrophy [71]. Moreover, low-carbohydrate, high-protein diets enhance changes in muscle strength and size and can increase energy expenditure [72, 73]. We recommend a low-calorie, high-protein diet, with 3 meals a day and postworkout shakes or healthy protein snacks. The exercise program is based on resistance training, such as free weights, resistance bands, and high-intensity interval training (HIIT). Once a week, a physical therapist should supervise the training to keep track of the activities and performance. There are no FDA-approved AOMs that improve muscle mass or increase energy expenditure. Phentermine, a noradrenergic appetite suppressant, may increase energy expenditure [74] and may stimulate thermogenesis by elevating norepinephrine levels and enhancing basal metabolic rate [75]. Thus, we recommended a combination of resistance training plus high-intensity interval training, with a potential support from phentermine.

#### Emotional eating phenotype

The emotional eating phenotype is characterized by negative mood and reward-seeking behaviors in relation to negative and positive emotions. These patients might benefit from a behavioral intervention structured for goal-setting, self-monitoring, and stimulus control [76]. We recommend intensive behavioral therapy with an emphasis on a high-intensity, cognitive behavioral program focused on identifying and changing emotional eating patterns while also building a support network and leaning positive coping skills [41]. We also favor the selection of naltrexone/bupropion sustained release as pharmacologic treatment. Wang et al [41, 77] showed that naltrexone-bupropion therapy attenuates brain activation in response to food cues in regions such as the hypothalamus while enhancing activation in areas involved in inhibitory control and internal awareness, suggesting control over eating behaviors by reducing the brain's reactivity to food signals and enhancing self-control mechanisms. This combination may help modulate appetite, mood, and cravings [5].

While newer AOMs such as glucagon-like peptide-1 receptor agonists and dual agonists are emerging as first-line therapies due to their efficacy, further research is needed to evaluate how these agents can be integrated into phenotype-based treatment approaches, building on lessons learned from earlier-generation pharmacotherapies

### Proof-of-Concept Studies for Phenotype-Tailored Obesity Therapy

When diets and lifestyle programs are compared against each other, the best predictor is the individual adherence to the caloric restriction [58, 78-80]. Thus, we tested our working hypothesis of phenotype-tailored lifestyle intervention (PhenoDiet) as described earlier and as summarized in Table 5. The PhenoDiet approach (diet, physical activity, exercise, and behavioral therapy) was studied in a pilot case-control study of 165 patients. Participants were predominantly women (90%) with a mean age (SD) of 43.9 (11.9) years, and a BMI of 38.3 (6.5). There were no statistically significant baseline differences between groups with regard to demographics, anthropometrics, or the metabolic variables analyzed. The PhenoDiet tailored approach was evaluated against the standard Mayo Clinic Diet in 84 and 81 participants, respectively. After 3 months, the phenotype-tailored lifestyle intervention resulted in a significantly greater weight loss of 7.4 kg (7%) compared to 4.3 kg (4%) in the standard care group. The proportion of patients who lost more than 5% at 3 months was 59% in the phenotype-tailored treated group compared with 33% in the control group, and the proportion of patients achieving a greater than 10% weight loss at 3 months was 26% in the phenotype-tailored treated group compared with 11% in controls [41].

**Table 5.** PhenoDiet treatment plan

Phenotype	Diet	Exercise and physical activity	Behavioral therapy
**Abnormal satiation**	• Volumetric, high-fiber, low-caloric diet; time-restricted eating (16:8); 1-2 meals a day	• >150 min per wk; 10 000 steps	• 12 sessions with behavioral coach
**Abnormal postprandial satiety**	• High-protein, low-calorie diet with premeal protein supplementation; 3-5 meals a day	• >150 min per wk; 10 000 steps	• 12 sessions with behavioral coach
**Abnormal emotional eating**	• Low-calorie diet with no snacks; 3 meals a day	• >150 min per wk; 10 000 steps	• Behavioral counseling; cognitive behavioral therapy weekly sessions
**Abnormal resting expenditure**	• Low-calorie diet with post workout protein supplementation; 3 meals a day	• Resistance plus high-intensity interval training (supervise once a week); 10 000 steps	• 12 sessions with behavioral coach

This table categorizes lifestyle interventions for obesity phenotypes during a 12-week, single-center clinical trial by Cifuentes (2023) [41].

**Figure 3.**
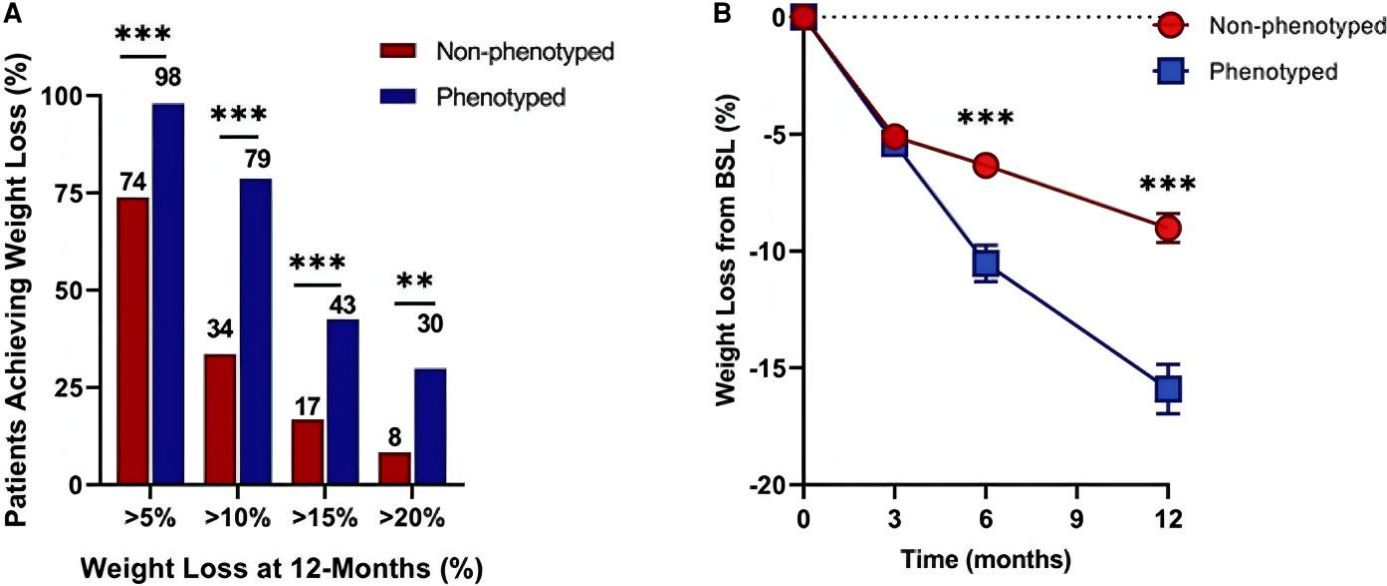
Phenotype-guided medications for obesity management improves weight loss outcomes. A) Percentage of patients achieving levels of weight loss after 1 year of non–phenotype-guided (n = 228) or phenotype-guided (n = 84) treatment. B) The percentage of total body weight loss from baseline in non–phenotype-guided and phenotype-guided treatment at 3, 6, and 12 months. **P less than .01; *** *P* less than .001. Retrieved from Acosta et al [40].

Our working hypothesis for phenotype-guided AOM selection is based on the following as described earlier and in Table 4. The working hypothesis was studied in a 12-month, real-world pragmatic trial performed at our Weight Management Center. In this study, 312 patients were assigned to phenotype-guided treatment or non–phenotype-guided treatment with AOMs (phentermine, phentermine/topiramate, bupropion/naltrexone, lorcaserin [no longer FDA approved] and liraglutide) as outlined earlier. Participants were predominantly middle age (±SD) (age 47 ± 5 years) White (96%) women (73%) with a BMI of 42 ± 1. Patients in the phenotype-guided group were younger and had lower systolic blood pressure and lower fasting glucose compared with the non–phenotype-guided group. There were no statistically significant baseline differences in sex, race, or anthropometrics. The phenotype-guided approach was associated with a 1.75-fold greater weight loss after 12 months with a mean weight loss of 15.9% compared to 9.0% in the non–phenotype-guided group. The proportion of patients who lost more than 10% at 12 months was 79% in the phenotype-guided group compared to 34% in the non–phenotype-guided treatment group (Fig. 3) [40]. We concluded that pathophysiological and behavioral phenotypes elucidate human obesity heterogeneity and can be targeted with AOMs to enhance weight loss. While the “real-world” clinical data support this approach, these results have performance and education biases. Thus, there is a need to pursue the highest level of evidence with a randomized, active-controlled, double-blind trial.

### Multi-omics

**Figure 4.**
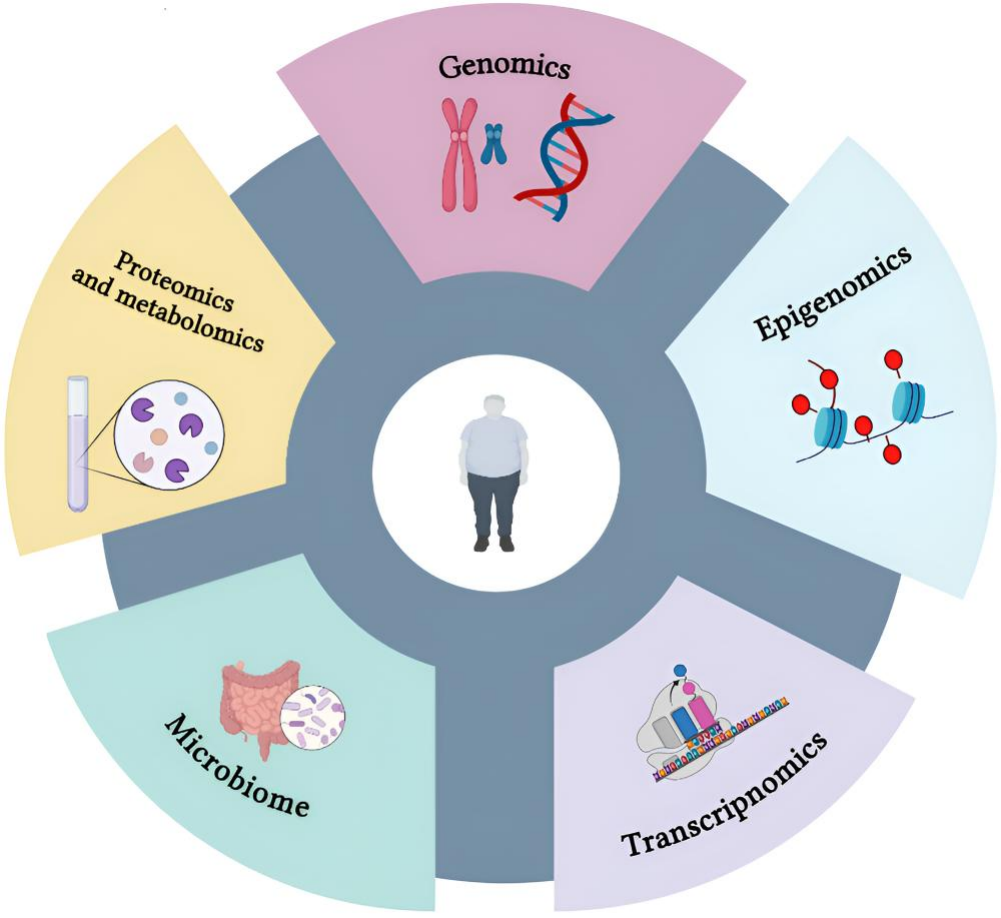
Multi-omics and precision medicine. Multi-omics integrates data from genomics, proteomics, metabolomics, and other molecular levels to provide a comprehensive understanding of health. Precision medicine uses this multi-omics approach to tailor treatments based on individual biological profiles. Together, they aim to optimize personalized care by targeting the root causes of diseases, such as obesity.

Obesity arises from a complex interplay of environmental and genetic factors [1]. This complexity leads to diverse clinical presentations and variable responses to weight-loss interventions. Multi-omics offers a cutting-edge approach to elucidating obesity's pathophysiology and enhancing obesity classification by integrating genomics, transcriptomics, proteomics, metabolomics, epigenomics, and microbiomics (Fig. 4). Current research focuses on combining these -omics biomarkers to refine phenotype classification and develop targeted personalized strategies for obesity management [81].

#### Genomics

Based on the genetic contribution, obesity can be categorized into two major types: monogenic obesity and polygenic common obesity. Monogenic obesity is caused by mutations predominantly in the leptin-melanocortin system, such as *LEP*, *LEPR*, *POMC*, and *MC4R* genes [82]*. MC4R* is the most common form of monogenic obesity [83 ], with variants found in around 5% of patients with obesity [84]. Targeted treatment options for monogenic obesity include pharmacologic interventions such as setmelanotide, an MC4R agonist, and metreleptin for congenital leptin deficiency. In 2020, the FDA approved setmelanotide (Imcivree) for chronic weight management in patients with obesity due to *POMC*, *PCSK1*, or *LEPR* deficiency [85]. In a phase 3 trial, 8 of 10 patients with *POMC* deficiency and 5 of 11 patients with *LEPR* deficiency lost at least 10% of their baseline body weight after 1 year of treatment with setmelanotide [86].

Most obesity cases are polygenic, involving multiple genes contributing to body weight regulation [87, 88]. The advent of genome-wide association studies (GWAS) further accelerated gene discovery. For instance, a recent combined GWAS meta-analysis identified 941 near-independent single-nucleotide variations (SNVs, formerly single-nucleotide polymorphisms) associated with BMI, explaining 6.0% of BMI variance in an independent sample [89].

Polygenic risk scores aggregate the effects of multiple genetic variants across the genome to estimate predisposition to obesity. Scores are usually calculated by summing the risk alleles weighted by their effect sizes from GWAS. Polygenic risk scores can be associated with obesity-related disease or obesity trajectory [90]. By identifying individuals at higher genetic risk for obesity, early intervention and prevention can be incorporated. Advances in genomics hold promise to inform the effectiveness of weight-loss interventions and help elucidate the genetic architecture of obesity by highlighting the cumulative effect of multiple SNVs. This understanding can guide the development of new therapeutic targets and interventions.

#### Transcriptomics

Transcriptomics analyzes RNA molecules to study gene expression patterns, offering insights into obesity-related mechanisms [91], including coding and noncoding messenger RNA. By identifying specific expressions of genes and their role in lipid metabolism, inflammation, and mitochondrial function, transcriptomics helps classify obesity subtypes and predict best response to treatments [92, 93].

Recent advances in transcriptomics have provided insights into obesity treatment. A study by Liu et al (2024) [94] comparing different weight-loss interventions, such as intermittent fasting (IF), exercise, and dietary modification, showed that each one induced unique transcriptomic signatures in subcutaneous fat, brown fat, skeletal muscles, and liver. These interventions differentially affect metabolic pathways, with IF prominently influencing genes involved in mitophagy and autophagy in adipose tissues and exercise broadly regulating the tricarboxylic acid cycle, carbon metabolism, and thermogenesis.

While progress has been made in transcriptome studies, research on therapeutic targets and applications is needed to develop effective treatment strategies for obesity.

#### Epigenomics

Epigenetics studies chemical modifications to DNA and associated proteins that regulate gene expression [95]. Mechanisms like DNA methylation play a key role in obesity's development by silencing genes and altering metabolic pathways [96]. Studies have linked methylation of genes involved in glucose metabolism, adipogenesis, and inflammation to obesity [97]. For instance, hypomethylation of the *ADRB3* gene was associated with an increased WHR [98]. Another study assessed methylation changes after a hypocaloric diet in normal-weight individuals, patients with obesity, and patients with a history of bariatric surgery. After this intervention, interleukin-6 methylation was increased after energy restriction and that was decreased in the bariatric surgery group [99]. This highlights the potential of epigenetic patterns in DNA, how they behave differently, and their potential as biomarkers for obesity.

While substantial progress has been made in epigenetic mechanisms underlying obesity, further research is needed to develop effective treatment strategies focused on epigenomics.

#### Proteomics

Proteomics analyzes the complete set of proteins to identify biomarkers related to obesity and weight loss [100]. For example, the DioGENES project identified 39 proteins, including well-known markers like adiponectin and C-reactive protein and novel candidates such as PRAP1 and D109 antigens associated with weight loss and maintenance [101]. Another study compared the plasma proteomes of 2 large independent cohorts among 1002 individuals with obesity and found an association of several proteins with BMI, with C-reactive protein showing the strongest association [102].

While genomics can identify SNVs associated with obesity, proteomics may reveal how these genetic variants affect protein expression and function [103]. These associations can aid in identifying new drug targets for treating obesity. However, most proteomic analyses of obesity have been limited by small sample sizes or a limited number of measured proteins [104]. Therefore, research involving large samples looking into associations between proteomics and obesity is promising.

#### Metabolomics

Metabolomics provides insights into obesity by quantifying metabolites and defining metabolic phenotypes (metabotypes) based on distinct biochemical signatures [105]. These signatures can guide personalized interventions. While large studies like DIETFITS and Food4Me failed to show significant gene-diet interactions, reanalysis of trials like DiOGenes and NUGENOB highlighted metabotype-specific dietary responses [106-110]. For example, individuals with normoglycemia benefited most from low-fat, high-carbohydrate diets, whereas those with prediabetes responded better to low-glycemic index diets [111].

Additionally, a study by Beyene et al [112] showed that metabolome-inferred BMI captured information on metabolic dysregulation independent of the measured BMI. These findings suggest the potential of the human metabolome to characterize the heterogeneity in obesity and identify individuals at increased risk of obesity-related diseases.

#### Microbiomics

Gut microbiota significantly influences nutrient absorption, energy regulation, and obesity development [113]. Gut microbiota composition can help predict responses to dietary interventions [114]. For example, individuals with a higher vs lower gut *Prevotella* to *Bacteroides* ratio lost significantly more weight on a calorie-restricted, high-fiber diet [115]. In addition, Jardon et al (2022) [116] discussed how the gut microbiome interacts with dietary macronutrients to influence host metabolism. They noted that the balance between carbohydrate and protein fermentation by gut microbiota and the site of fermentation in the colon are critical determinants of metabolic health.

Future research in this field could help determine microbiome profiling to predict response to dietary, pharmacologic, and surgical interventions, advancing the field of precision medicine for obesity.

## Integration of Multi-omics Data and Precision Medicine for Obesity

The integration of multi-omics data shows potential in obesity classification and future management. Watanabe et al [117] proposed that using “biological BMI” machine-learning model predictions calculated from multi-omics data and clinical laboratory tests from blood reflected metabolic health more accurately and responded more to lifestyle changes than classically measured BMI. In addition, a score that is more responsive to lifestyle changes can provide valuable feedback on and monitor the effect of health interventions [118].

Integrating multi-omics data into predictive models for response to treatment remains challenging. Comprehensive multi-omics and big data analysis are outside this review's scope and are covered by Woldemariam et al [81]. In addition, the development of omics-based biomarkers requires large prospective cohorts for training and validation, particularly for the measurement of phenotypic such as parameters of energy balance and appetite-regulation traits that are not frequently measured in biobanks. Finally, limited sample sizes in genomic studies present a considerable limitation that warrants attention in future investigations.

## Conclusion

The traditional “one-size-fits-all” approach to obesity treatment often falls short when addressing the condition’s complexity and variability. It frequently shows inconsistent responses and heterogeneous outcomes. With obesity prevalence rising, the need for more precise and effective strategies is imperative.

Individualizing treatment by considering obesity pathophysiology and parameters of energy balance regulation shows great promise, as mechanistic studies could inform treatment selection based on an individual’s major trait. However, this approach is expensive and time-consuming. Developing omics-based biomarkers could be key to enabling the broader adoption of a precision medicine framework for weight-loss interventions.

Precision medicine offers a transformative solution by tailoring treatments to individual profiles using insights from advanced -omics technologies. These tools have expanded our understanding of obesity's multifactorial etiology, progression, and treatment responses, paving the way for more personalized therapies.

## Data Availability

Data sharing is not applicable to this article as no data sets were generated or analyzed during the current study.

## Abbreviations

AACE: American Association of Clinical Endocrinologists
ACC: American College of Cardiology
ACE: American College of Endocrinology
AHA: American Heart Association
AOM: antiobesity medication
BMI: body mass index
CVD: cardiovascular disease
EOSS: Edmonton Obesity Staging System
ER: extended release
FDA: US Food and Drug Administration
GWAS: genome-wide association studies
IF: intermittent fasting
MHO: metabolically healthy obesity
MUO: metabolically unhealthy obesity
TOS: The Obesity Society
WC: waist circumference
WHR: waist-hip ratio
